# Factors contributing to attrition behavior in diabetes self-management programs: A mixed method approach

**DOI:** 10.1186/1472-6963-8-33

**Published:** 2008-02-04

**Authors:** Enza Gucciardi, Margaret DeMelo, Ana Offenheim, Donna E Stewart

**Affiliations:** 1 Ryerson University, School of Nutrition, Toronto, Ontario, Canada; 2 University Health Network, Women's Health Program Toronto, Ontario, Canada; 3 University Health Network, Diabetes Education Centre, Toronto, Ontario, Canada; 4 University of Toronto, Faculty of Medicine, Toronto, Ontario, Canada

## Abstract

**Background:**

Diabetes self-management education is a critical component in diabetes care. Despite worldwide efforts to develop efficacious DSME programs, high attrition rates are often reported in clinical practice. The objective of this study was to examine factors that may contribute to attrition behavior in diabetes self-management programs.

**Methods:**

We conducted telephone interviews with individuals who had Type 2 diabetes (n = 267) and attended a diabetes education centre. Multivariable logistic regression was performed to identify factors associated with attrition behavior. Forty-four percent of participants (n = 118) withdrew prematurely from the program and were asked an open-ended question regarding their discontinuation of services. We used content analysis to code and generate themes, which were then organized under the Behavioral Model of Health Service Utilization.

**Results:**

Working full and part-time, being over 65 years of age, having a regular primary care physician or fewer diabetes symptoms were contributing factors to attrition behaviour in our multivariable logistic regression. The most common reasons given by participants for attrition from the program were conflict between their work schedules and the centre's hours of operation, patients' confidence in their own knowledge and ability when managing their diabetes, apathy towards diabetes education, distance to the centre, forgetfulness, regular physician consultation, low perceived seriousness of diabetes, and lack of familiarity with the centre and its services. There was considerable overlap between our quantitative and qualitative results.

**Conclusion:**

Reducing attrition behaviour requires a range of strategies targeted towards delivering convenient and accessible services, familiarizing individuals with these services, increasing communication between centres and their patients, and creating better partnerships between centres and primary care physicians.

## Background

Diabetes self-management education (DSME) has emerged as a resource to assist individuals in actively participating in their diabetes care. The goals of DSME are to help individuals modify lifestyle behaviors, optimize glycemic control, and to prevent acute and chronic complications. Given that diabetes management is a life-long endeavor, a key component of DSME, or any chronic disease self-management program, is ongoing follow-up and continued assistance with management needs [1,2]. According to a meta-analyses by Norris et al., (2002) greater contact time between educator and patient was the only statistically and clinically significant predictor of improved glycemic control in DSME interventions [3]. In meta-regression by Ellis et al., (2006), face-to-face delivery, cognitive re-framing, and exercise content were reported as statistically and clinically significant predictors of improved glycemic control in DSME interventions [4]. Although these interventions have proven to be efficacious [3,5], they have been observed to have high attrition [5], particularly in clinical practice [6]. Individuals who drop out of DSME adhere less conscientiously to self-management activities and have worse glycemic control and health outcomes than those who continue with the recommended education and follow-up [7-12]. However, little and inconsistent information is available about factors linked with attrition behavior in DSME interventions [6,58].

Even though it is now well recognized that success or failure of any education program may depend on individuals' initial attitudes or beliefs [13], the majority of the studies examining program attrition focus primarily on sociodemographics and clinical factors, paying little attention to psychosocial and contextual factors that may influence attrition behavior. Furthermore, individuals themselves are an important resource in better understanding attrition behavior, which is seldom represented in the literature [6].

Andersen's Behavioral Model of Health Services Utilization is a theoretical model that has been used to explore individual and contextual characteristics that may facilitate or impede health services utilization in a much broader orientation than most other models [14,15]. The model proposes that health behaviors are influenced by individual characteristics that can be divided into the following categories: predisposing, enabling and need factors. Predisposing factors are existing conditions (e.g., sex, age, and psychosocial factors such as attitudes and beliefs etc.) that may influence or predispose the use of health services. Enabling factors include personal, family, and community resources that can either facilitate or impede the use of services. Need factors refer to the conditions (e.g., functional capacity, symptoms, and general health status) perceived by laypeople or evaluated by health care providers as requiring medical treatment or use of health services. Andersen's model has been extensively used to explain or predict the use of many different health services [16,17] such as mammography, breast self-exams [18], cardiac rehabilitation programs [19], influenza vaccination [20], home care services [21], mental health services [22], occupational therapy services [23], support groups [24], children's medical services [25], publicly funded services [26], physician care [27], usual sources of care [28] and hospitalization [29]. Subjects in these studies have included children, adults and older adults, urban and rural residents, people from diverse ethnic and racial backgrounds and individuals with or without specific illnesses. This model has also been adapted to explain or predict access to and use of medication [30], receipt of disease-prevention advice from physicians [31] and quality of life issues [32]. Although this model has never been used to explain use or attrition of DSME, we thought this model would be the most appropriate to elucidate potential factors contributing to attrition from DSME.

Considering the amount of time and money invested in the planning and structuring of self-management education programs, it is imperative to understand why people choose to engage or disengage in education initiatives in order to achieve program effectiveness. The objective of this study is to investigate factors that may contribute to attrition behavior in DSME using both quantitative and qualitative approaches. The Behavioral Model of Health Service Utilization will be used to organize themes and domains that emerge from the qualitative data.

## Methods

### Research Settings

This study was conducted at a diabetes education centre (DEC), located within a hospital that serves one of the most culturally diverse urban communities in Toronto, Canada. The DEC provides initial one-on-one health assessments with a dietitian and nurse, whereby patients and educators work together to develop self-management goals and a nutritional care plan. Patients are recommended to return and attend individual counseling or classes delivered by a multidisciplinary health care team over three consecutive days (15 hrs). Patients are also offered ongoing individual or group follow-up visits, giving them the opportunity to discuss their self-management activities and potential concerns with educators, and allowing educators to monitor patient progress.

During individual counselling and group education classes, educators address and are responsive to patients' cultural customs, values, beliefs and literacy levels. Family members are welcomed and accommodations are made to include them if a patient's wishes to do so. A predominant use of visual aids, low literacy level education resources, and hands-on interactive activities help to facilitate skill development. During individual visits, educators reassess patients' status and identify their priorities. The most common course for follow-up is face-to-face communication with the same diabetes educators. Situational problem-solving strategies are used to enable patients to cope with challenging social settings. Group classes include approximately five to eight patients. The various teaching methods include didactic methods, mutual goal setting, situational problem solving, cognitive reframing, interactive sensory-stimulating and role-playing methods.

The DEC hours of operation are from 8:30 a.m. to 4:00 p.m. Patients are usually referred to the centre by their primary care physician or endocrinologist, but a referral is not required. These services do not incur a direct cost to patients as they are covered by provincial health insurance. All services, including classes, are provided in six different languages by bilingual staff or professional interpreters.

### Research Subjects and Design

Individuals with Type 2 diabetes who were initially assessed at the centre between August 1, 2000 and July 31, 2001, and who primarily spoke English, Portuguese, or Cantonese, were invited to participate in a telephone interview in August 2002. These three language groups represent the largest patient-populations of the centre. Interviews were conducted in these three languages.

Due to the high proportion of English-speaking patients, we systematically selected every second patient (50%) from an English-speaking alphabetical sampling list, yielding a more equitable distribution of language groups for the analyses. The sampling fraction for each of the non-English speaking groups was 100%. Those with a diagnosis of gestational diabetes, impaired glucose tolerance, an unstable psychiatric condition or those assessed by an educator as not requiring diabetes education as noted in their medical charts were excluded from the study. A maximum of 10 calls per individual were made at different times of the day and week to recruit participants. Of 399 patients, we were able to contact 309, of whom 267 agreed to participate in our interview (participation rate of 86%). Of those we were unable to contact, 11% were inaccessible by phone, 9% had changed phone numbers, and 3% were deceased. In order to gain knowledge and understanding of attrition behavior from the patients' perspective, we asked participants who prematurely withdrew from the program their reasons for discontinuing the use of DSME services using an open-ended question. Only one question was asked to reduce patient burden and to increase our response rate. The institution's research ethics board approved the study.

## Analysis

### Outcome variable

For the most part, DSME is individualized for each patient based on the severity of the illness and the perceived need for care. There are no standard recommendations for the number of educator visits or requisite contact hours for optimal diabetes management. Consequently, a standard method of measuring attrition behavior in DSME is difficult to define and currently nonexistent. In collaboration with a panel of program managers and educators, we approximated a minimum level of participation that may ensure satisfactory knowledge and skills to carry out diabetes self-management activities based on the fundamental principles of DSME (i.e., initial assessment, education, and follow-up/evaluation), as defined by the American Diabetes Clinical Practice Guidelines [33]. The panel came to a consensus that four educator contacts was the minimum number of contacts required to cover the basic components of DSME over a one-year period. It was argued that patients should ideally have an initial health assessment with a nurse and dietitian educator, some formal education either through counseling or classes, and a follow-up visit for evaluation of self-management practices. For our analyses, attrition was defined as not returning to the centre for at least four educator contacts over a one-year period. After and including the first contact, study participants were classified as users (= 4 educator contacts) or nonusers (≤ 3 educator contacts) of ongoing DSME services. An educator contact was described as a nurse/dietitian assessment, participation in an education class or nurse/dietitian counseling, or a group or nurse/dietitian follow-up. More than one educator contact could be achieved in one visit. Medical chart information on service utilization at the centre was validated with participants at the beginning of their telephone interviews.

### Independent Variables

Independent variables were selected based on a review of previously published bivariate and multivariable analyses [34] in the area of attrition or missed appointments from diabetes education programs [58]. Association between attrition and the following variables were examined: sex, age (≤ 49, 50–64, ≥ 65), patients' primary language spoken (English, Cantonese and Portuguese), marital status (single, never married, divorced or widowed, and married or living with partner), education (< grade 9 and some high school or more), employment status (employed full or part-time, unemployed and retired), household income (continuous), having a regular physician (yes and no) and the summation of the number of diabetes-related symptoms at the time of first visit as reported by a diabetes educator in the patients' medical record during assessment (based on a checklist with designated space for other symptoms not listed).

### Quantitative Analyses

Independent variables were compared between users and nonusers of ongoing services by employing Pearson's Chi-square statistic for categorical variables and Independent t-tests or Mann-Whitney tests (skewed distributions) for continuous variables. Bivariate and multivariable logistic regression analyses were used to identify predictors of attrition. The full multivariable model adjusted for all independent variables was fitted [34]. Our sample size calculation [35] (pg. 91) suggested that 171 participants were required for a two-sided 95% confidence interval with a total width of 0.15 for an expected proportion (i.e., attrition rate) of 50% [36]. Our sample size was sufficient to fit a multivariable model with 12 parameters based on a widely used rule of thumb of approximately 10 events per parameter in order to obtain reliable estimates in logistic regression [37]. With two exceptions, all variables had less than 5% missing data; therefore, we used single imputation, replacing missing continuous data with means and missing categorical data with modes [38]. The two exceptions were education and household income. We replaced missing education data with mode neighborhood education levels based on participant's postal codes and data from the 2001 Canada Census. We also replaced missing household income data (46.4%) with median neighborhood income levels based on a participant's postal codes and data from the 2001 Canada Census. This approach has previously been shown to be valid [39]. SPSS version 12 was used in to conduct these analyses [40].

### Qualitative Analyses

Responses to the open-ended question were analyzed using conventional content analysis [41]. We created 126 unique codes, which represented reasons for program attrition. To be considered a code, a topic had to be mentioned by more than one study participant. The first author (E.G.) developed the initial codes, and the second author (D.M.) reviewed the coding scheme for logic and breadth. Any disagreements were resolved through further discussion. The third (A.O.) and fourth author (D.S.) independently coded the responses using the coding scheme. The kappa statistic was performed to examine the inter-coder reliability for concordance in how the text was coded in SPSS. Coding agreement between two raters was 94%. The codes were later organized into themes and further into broader domains. Responses were analyzed, coded, and categorized using QSR 6 NUDIST [42]. To confirm our key findings, our themes and domains were later organized under predisposing, enabling, and need factors.

## Results

### Quantitative Results

Characteristics of the study population are presented in Table 1. There was an equitable mix of men and women (53.9%) in the study population; the average age was 57.9 years, the majority of the sample was married or living with a partner (73%), and the median time living with diabetes was 3 years. The median household income of our study population was $47,234.00 (CAD) considerably less than the median household income for Ontarian ($79,697) and Canadian families ($72,524) in 2000. Participants were more likely to be referred to the centre by their primary care physician (47.7%) and endocrinologist (27.3%). Among participants, 44.2% (n = 118) were classified as nonusers of ongoing services. Study participants were younger, and had diabetes for fewer years than those who did not participate (65.0 years, standard deviation 11.94; 7 years, inter-quartile range 1.5–15). There was no statistically significant difference in user status among study participants (55.8%) and non-participants (49.0%).

**Table 1:** Characteristics of research participants by users and nonusers of ongoing services

Characteristic	Overall (N = 267) % (n) or Mean (± SD)	Users (n = 149) % or Mean (± SD)	Nonusers (n = 118) % or Mean (± SD)	P-value
Sex				0.163
Male	46.1 (123)	42.3	50.8	
Female	53.9 (144)	57.7	49.2	
Age continuous (years)	57.9 (± 11.8)	58.66 (± 10.9)	56.80 (± 12.8)	0.180
Age categories				0.035
≤ 49	24.3 (65)	18.8	31.4	
50–64	45.3 (121)	51.0	38.1	
≥ 65	30.3 (81)	30.2	30.5	
Primary language spoken				0.000
English	42.3 (113)	31.5	55.9	
Cantonese	39.3 (105)	47.0	29.7	
Portuguese	18.4 (49)	21.5	14.4	
Marital status				0.074
Single/never married/divorced/widowed	27.0 (70)	23.3	31.9	
Married/living with partner	73.0 (189)	76.7	68.1	
Education				0.002
< Grade 9	48.3 (128)	56.8	43.2	
Some high school and up	51.7 (137)	37.6	46.7	
Household Income	48,900 (± 15,400)	49,000 (± 12,800)	48,800.00 (± 19,100)	0.070
Primary daily activity				0.000
Employed full or part-time	39.8 (106)	26.8	56.4	
Unemployed	32.0 (85)	39.6	22.2	
Retired	28.2 (75)	33.6	21.4	
Time since diagnosis (years)	3.0 (0.25, 10)^a^	4.0 (0.25, 10)^a^	3.0 (0.25, 10.0)^a^	0.56
Age of diagnosis (years)	51 (± 11.54)	52.38 (± 11.4)	50.96 (± 12.1)	0.34
Type of medical management				0.03
Medical nutrition therapy	25.4 (67)	19.5	33.0	
Oral agents	64.0 (169)	70.5	55.7	
Insulin	10.6 (28)	10.1	11.3	
Referral				0.895
Primary care physician	47.7 (126)	49.0	46.2	
Diabetes specialist/endocrinologist	27.3 (72)	27.2	27.4	
Self	4.2 (11)	3.4	5.1	
Other	20.8 (55)	20.4	21.4	
Have a regular primary care physician				0.019
Yes	87.7 (228)	83.4	93.0	
No	12.3 (32)	16.6	7.0	
Number of contacts	1.0 (0, 3.00)^a^	8.0 (5.0, 12.50)^a^	2.0 (1.0, 2.0)^a^	0.000
Number of diabetes symptoms	1.73 (0.0, 3.0)^a^	2.0 (0.0, 3.0)^a^	1.0 (0.0, 2.0)^a^	0.000
BMI (kg/m2)	30.78 (± 6.5)	30.7 (± 6.74)	30.9 (± 6.21)	0.793
Distance from patients' residence to centre in KM	5.6 (2.8, 13.1)^a^	5.25 (2.5, 10.0)^a^	6.9 (3.2,16.9)^a^	0.084

**Note: **Some percentages may not add up to 100% due to missing data; SD = Standard deviations;

^a^Median and interquartile range

The multivariable model including all independent variables adequately distinguished between users and nonusers of ongoing services (c statistic = 0.75 [CI, 0.70, 0.81]). The model also appeared to be well calibrated according the Hosmer-Lemeshow goodness of fit statistic (p-value = 0.95). The odds of attrition were higher amongst the elderly (OR 3.21, CI 1.46–7.06) compared to middle-aged participants, and higher among participants who had a regular primary care physician (OR 3.25, CI 1.27–8.32) compared to those who did not. The odds of attrition were lower for those who were unemployed (OR 0.25, CI 0.13–0.52) or retired (OR 0.23, CI 0.09 – 0.57) compared to those who were working full and part-time. Participants who experienced greater diabetes-related symptoms had lower odds of attrition (OR 0.78, CI 0.65–0.94) (see Table 2).

**Table 2:** Bivariate and multivariable logistic regression: predictive model of attrition from diabetes self-management education (N = 267)

	Nonuse of ongoing Diabetes Self-management Education (n = 267)			
Characteristic	Bivariate OR (95% CL)	P-value	Multivariable OR (95% CL)	P-value

Sex				
Male	1.00 (referent)		1.00 (referent)	
Female	0.71 (0.44–1.15)	0.164	0.90 (0.51–1.59)	0.719
Age		0.037		0.013
≤ 49	2.23 (1.21–4.12)	0.010	1.47 (0.72–3.00)	0.290
50–64	1.00 (referent)		1.00 (referent)	
≥ 65	1.35 (0.76–2.40)	0.303	3.21 (1.46–7.06)	0.004
Primary language spoken		0.000		0.133
English	1.00 (referent)		1.00 (referent)	
Cantonese	0.38 (0.19–0.76)	0.006	0.48 (0.20–1.14)	0.096
Portuguese	0.36 (0.21–0.62)	0.000	0.49 (0.22–1.10)	0.083
Marital status				
Single/never married/divorced/widowed	1.00 (referent)		1.00 (referent)	
Married/living with partner	0.65 (0.37–1.13)	0.125	1.22 (0.61–2.42)	0.574
Education				
< Grade 9	1.00 (referent)		1.00 (referent)	
Some high school and up	2.18 (1.33–3.58)	0.002	0.91 (0.45–1.84)	0.790
Employment		0.000		0.000
Full or part-time work (referent)	1.00 (referent)		1.00 (referent)	
Unemployed	0.27 (0.15–0.49)	0.000	0.25 (0.13–0.52)	0.000
Retired	0.30 (0.16–0.56)	0.000	0.23 (0.09 – 0.57)	0.002
Household income (units of $10,000)	1.00 (0.87–1.17)	0.905	0.983 (0.83–1.16)	0.844
Regular primary care physician				
Yes	2.65 (1.14–6.15)	0.024	3.25 (1.27–8.32)	0.014
No	1.00 (referent)		1.00 (referent)	
Diabetes symptoms at first visit	0.76 (0.65–0.89)	0.001	0.78 (0.65–0.94)	0.009

Note: The main measure of association used was the odds ratio (OR) with its 95% confidence interval (CI).

Participant's primary language and education was identified as a significant predictor of attrition at the bivariate, but not at multivariable level. Further examination revealed that employment status may be a mediator between primary language and use of ongoing services, and education and use of ongoing services. A significantly greater percentage of patients who primarily spoke English or who had a grade nine education or higher worked full or part-time compared to those who primarily spoke Portuguese or Cantonese or had less than a grade nine education. When we compared the log (odds ratios) and their standard errors separately for primary language and education without and with employment status in the model, the magnitude of the effect of primary language and education diminished; that is, the parameter estimates became smaller and the standard error remained fairly stable (data not shown). Therefore, we postulate that employment status is an intermediary variable, in that having English as one's primary language or an education level of grade nine or higher may enable one to get employment, but working full or part-time is a barrier to ongoing use of services.

### Qualitative Results

Of the 118 nonusers, 97 (82.2%) provided reasons for discontinuing their use of DSME services. Of the 126 codes created, 44.4% were categorized as predisposing factors, 49.3% as enabling factors, and 6.3% as need factors. Responses were similar across sex and language groups. The common factors, domains, and themes representing patient-identified reasons for attrition from DSME were summarized in Table 3.

**Table 3:** Summary of factors, domains, & themes contributing to program attrition (N = 97).

Factors	Domains	Major themes and their dimensions
Enabling	Availability of support	**Formal support**
		Conflict with centre's hours of operation
		Lack of an effective appointment reminder system
		Proximity to the centre
		Difficulties in locating the centre
		Regular physician visits
		**Informal support**
		Dependent on family assistance
		Family responsibilities
	Familiarity with resources	**Diabetes education centre and its services **
		Familiarity with DEC services
Predisposing	Self-efficacy	**Diabetes self-management**
		Sufficient amount of information
		Adherent to self-management activities
		Non-adherent to self-management activities
	Attitudes	**Diabetes education**
		Level of priority
		Apathy
	Co-morbidity	**Competing health conditions**
Need	Perceived/evaluated	**Disease severity**
		Patient judgment of severity
		Physician judgment of severity

Conflict between patients' work schedules and the centre's hours of operation was the most frequently cited response for attrition under the enabling category: "Those are inconvenient hours for me because of work" and "I can't take days off work." Several patients also reported not being reminded of their upcoming appointment by the centre and forgot to attend. Distance from the centre and trouble finding the centre were also reported as barriers. Some stated that seeing a specialist or their primary care physician regularly was sufficient: "I have check-ups with my doctor regularly," "I consulted the diabetes specialist in the hospital," and "I always talk to the doctor about diabetes". Regarding informal support, some participants reported not having family to accompany them to the centre, while others were too busy tending to family responsibilities such as caring for children or an elderly family member. Another common response for program attrition was the lack of familiarity with the DEC and its services: "I didn't know too much about the program," and "I didn't know I had to continue to come back."

Among predisposing factors, the majority of responses were associated with self-efficacy. Many patients articulated confidence in their knowledge and ability to manage their diabetes: "I have enough information," "I am well informed by my relative who has diabetes," "I have had diabetes for a long time," "My diabetes is under control," and "I am taking my medicine regularly." Conversely, a few patients failed to adhere to management recommendations and consequently felt embarrassed to return to the centre. Some also mentioned they sought out other sources of diabetes information: "I found information easier to obtain from other sources," "I have been given a book on food for diet," and "I researched on the net a lot." Others expressed apathy towards diabetes education or that diabetes education was not a leading priority, claiming that they "have a just leave it attitude," and were simply "too lazy" to return. Patients also stated that they were "too busy," "had no time," or had "other priorities during the day." A number of nonusers stated that having multiple health conditions was a barrier to returning to the centre: "I find it difficult to balance all my health problems" and "I am dealing with too many complications."

Under need factors, a few patients identified a low perceived seriousness or severity of their diabetes as the reason for not returning to the centre. Others stated that their physician said they had "borderline" or "mild diabetes," and that their diabetes "is not too serious."

## Discussion

### Enabling factors

Although it is ultimately an individual's decision to seek help and comply with the education recommendations, various facets within the healthcare system can influence their behavior. Our findings suggest that many factors contributing to attrition behavior are rooted in how services are structured and delivered. For instance, conflict between work schedules and the centre's hours of operation was the most cited reason for attrition. In addition to a few descriptive studies [11,12], our quantitative results corroborate this finding suggesting that those who work full or part-time are more likely to abandon DSME programs prematurely than those who are unemployed or retired. Participating in education programs can be time intensive and costly for patients, their family members or friends involved in their care. Offering education services that are less intensive and during convenient hours that fit into individuals' schedules may increase access and ongoing use of these services [43]. Programs that are flexible and offer a range of education options via internet, mail, or telephone may also reach and retain more working individuals [44]. For instance, telephone calls have cost and logistic advantages that are only beginning to be appreciated. Wasson et al. showed that substituting regularly scheduled follow-up phone calls for irregular follow-up visits substantially improved health status and reduced costs for chronically ill patients [45]. Alternative methods of delivering education, such as telephone and home visits may also alleviate many of the access barriers faced by the elderly, individuals who are caring for children or family members, or those who need assistance in attending education programs.

Establishing communication and an exchange of information between centres and patients is recommended right from the point of referral. For instance, some of our participants reported not being well informed about the centre and their services, and were unaware of the expectations in attending. Although referring physicians may provide patients with some information regarding local diabetes resources, DECs should provide information about their services directly to patients prior to their first visit. Diabetes clinics that provide patients with information on when and where to go, where to park, what to bring, whom they will see, and what to expect, in addition to providing a reminder call prior to the appointment, dramatically reduce initial non-attendance rate [46]. This comprehensive approach may also reduce instances of loss to follow-up by equipping patients with the knowledge necessary to navigate these services and to understand the centre's goals and expectations. Adequately informing and orientating patients about DSME services may be an important factor in retaining patients in the program.

Primary care physicians are mainly responsible for the management of this complex disease. They are an important source of information, motivation, and encouragement that can enhance the use, and thereby the effectiveness, of behavioural programs such as DSME. In fact, care providers' enthusiasm for preventative health programs appears to influence their patients' utilization [47]. Therefore, it is surprising to observe that having a regular primary care physician is associated with attrition behavior in both our qualitative and quantitative results. Some participants perceive that specialists or primary care physicians provide the same coverage of diabetes education and lifestyle modification skills training as DECs. Similar results were reported by Lloyd et al., where a number of missed diabetes clinic appointments were a result of seeing a diabetes specialist or a family physician regularly as remarked by patients [48]. Primary care physicians need to support and encourage their patients to fully participate in DSME, as an adjunct to their services rather than a repetitive or competitive service. Fear of losing patients to DECs should be alleviated through better communication and partnership between centres and primary care physicians.

### Predisposing Factors

Among predisposing factors, the majority of responses regarding program attrition stems from participants' perceived confidence in the amount of diabetes knowledge already acquired and a high level of self-efficacy in their ability to adhere to self-care activities. It has been noted in the literature that some individuals may fail to recognize the additional benefits of DSME due to an overestimation of personal knowledge and competency in self-care activities [49,50]. However, longitudinal assessment of the degree of DSME utilization and its affect on knowledge, self-care behaviors, and health outcomes is needed to substantiate this postulation. In the mean time, diabetes educators should assess patients' knowledge, including education provided by primary care physicians or during hospitalization, to complement and enhance each patient's knowledge base, and to avoid redundancy [51].

Conversely, inability to adhere to management recommendations can cause embarrassment and lead to program attrition as reported by some of our participants and other studies. For instance, negative outcomes such as poor glycemic control or relapse in glycemic control increases the likelihood of patients dropping out from a diabetes clinic [10]. Individuals with diabetes are more reluctant to revisit their dietitian if their blood sugars were too high or if their weight did not change [52]. Dropping out of a weight-loss program for those with Type 2 diabetes was also significantly associated with weight gain at the last follow-up visit [53]. Educators should help patients set realistic management goals, stress the challenges of lifestyle modifications, the possibilities of relapse, and allay feelings of embarrassment or fear. Educators need to take a more patient-centre approach to counseling and care, to ensure that they are non-judgmental, actively listening to patients and empowering them to make their own decisions.

Other predisposing variables that seem to influence attrition behavior are apathy and a low priority attitude towards diabetes education. Similar findings are observed in the literature, where individuals express lack of time as a reason for missing dietitian appointments [52,54] or diabetes clinic visits [12]; while Graziani et al. report patient apathy (17%) and patients' being too busy (29%) to attend diabetes education classes [54]. These findings illustrate two points: either DSME services are offered at inconvenient times or DSME has been given a low-priority placement by patients with Type 2 diabetes. To further elaborate on the latter point, inadequate uptake of DSME may still reflect a culture that perceives Type 2 to be the 'mild' form of the disease, despite its high morbidity and mortality rates [55] or may reflect a cultural perspective whereby developing expertise is not yet valued, highlighting the need for a paradigm shift within the healthcare system and in patients to enhance personal responsibility and a sense of control over one's chronic illness [55]. A better understanding of patients' perceptions of their role in the management and care of their illness may provide some insight on who should be targeted for DSME.

### Need Factors

Type 2 diabetes can initially be asymptomatic and progress slowly, which may cause the observed apathy and low priority attitude towards diabetes education. Newly diagnosed individuals may take months or even years to come to grips with the serious health threat posed by diabetes [56]. Deteriorating health or major life events are most often the impetus for patients to make significant lifestyle changes. Once individuals reach this point, they are more likely to perceive diabetes as a serious illness and seek professional advice, participate in diabetes education, and intensify treatment of their diabetes [56]. This may also explain why our participants presenting with greater diabetes-related symptoms are more likely to be users of ongoing services. Likewise, Simmons et al (2000) observed that non-attendees (i.e., patients who missed three quarterly appointments) were less likely to have been diagnosed with diabetes-related symptoms than attendees [57]. Efforts are required to encourage patients with fewer diabetes symptoms not to abandon DSME and to better educate them on the potential diabetes-related complications [49].

A care provider's evaluation of a patient's condition (i.e., evaluated severity) may also influence the patient's decision to prematurely disengage in DSME. For instance, the physician's reaction at the time of diagnosis may affect a patient's attitude toward the disease, use of DSME, and adherence to self-care behaviors. Therefore, physicians framing diabetes as 'borderline' or 'mild', as stated by some of our study participants, may decrease a patient's sense of urgency to manage their diabetes aggressively. Inconsequential attitudes towards diabetes and self-management training can undermine individuals' motivations, and potentially hinder maintenance of diabetes self-care. Both evaluated and perceived severity of diabetes can be modified through clinical practice guidelines and public health awareness campaigns [14].

The attrition rate in this study was 44.2%. One would think that attrition rates within subsidized health care systems such as that in Canada may be lower than in a fee for service type health care system. However, this may not necessarily be the case. Based on a systematic literature review on attrition from diabetes education services [58], a wide-ranging proportion of individuals with diabetes drop out of education interventions across several countries with differing structured health care systems. The reported attrition rates from diabetes education services in Britain range from 4% to 19% [7,12,48,59], while rates are higher in the United States ranging from 12% to 50 % [10,11,60] and in Japan ranging from 35% to 57% [8,61]. Attrition rates are also higher in other countries such as, Ireland (41%) [62] and Canada (50%) [58]. Given the implications on cost, program effectiveness, and the inability to meet the needs of some people with diabetes, attrition is undoubtedly a concern across health care systems.

### Application of the Theoretical Model Used

The application of Andersen's Behavioral Model of Health Services Utilization, as reported in the literature, has been limited to disease care with very few empirical applications to secondary prevention. Given our findings however, the model shows promise in predicting the use of secondary preventative health services and in identifying priority areas to improve access to these interventions. The majority of reasons for abandoning DSME were of the predisposing and enabling kind, suggesting that both personal and contextual factors affect attrition behavior. Traditionally, attempts to change behaviour have focused on the individual, yet our study findings recommend expanding the focus from the individual to broader determinants. Therefore, a comprehensive approach to health promotion also requires changing social system practices that affect health behaviour.

According to this framework, inequitable access to services occur when sociodemographics and enabling resources determine whether or not, or how much care is available or sought rather than need factors [15]. Our findings identify disparities in access to DSME for those who are elderly, work during standard work hours, and for those who are primary caregivers. Fortunately, accessibility and delivery of DSME are modifiable factors, and their alteration to meet patient needs often leads to better service utilization [14]. Future research is needed to explore how best to deliver educational services to increase access and retention behavior in these programs.

The strength of this study lies in its multiple-methods approach and the considerable overlap between our quantitative and qualitative results. However, there are some limitations that should be considered. As this was an exploratory examination of aspects contributing to attrition, many factors that can potentially affect attrition behavior were not measured. For instance, we cannot rule out the characteristics of referring physicians or educators, organizational factors, or education delivery and content that may have an impact on patient satisfaction and the desire to revisit the centre. This topic is a complex phenomenon, but obtaining first-hand information from patients regarding their use of these services is a good starting point. It is also possible that study participants may have underreported some issues as we used a brief open-ended question to assess reasons for attrition. Nevertheless, our interviewers made it very clear that they were independent from and not employees of the centre, to ease any discomfort of addressing negative issues about the DEC. Factors affecting attendance are likely to differ across organizations due to the heterogeneous nature of DSME programs. Therefore, similar research in different locales is needed to confirm or refute our results. Future research should employ a prospective research design, which can provide causal inferences of program attrition, rather than relying on patient recall in retrospective or cross-sectional studies.

## Conclusion

By using a mixed-method approach, we gained an in-depth understanding of both personal and contextual factors contributing to attrition behavior in DSME programs. Although a few of these issues have been identified by previous research, there is still a gap between what is recommended and what is currently supported and practiced in clinical settings. Tackling program attrition will inevitably involve the implementation of multiple strategies to address the numerous barriers that exist for different groups of individuals. Our findings suggest that reducing attrition behaviour requires strategies targeted towards delivering convenient and accessible services, familiarizing patients with these services, better informing patients about the severity and potential complications of diabetes, enhancing communication between DECs and their patients, and creating better partnerships between centres and primary care physicians. Restructuring DSME should yield programs that offer greater access, convenience and choice for those with diabetes.

## Competing interests

The author(s) declare that they have no competing interests.

## Authors' contributions

EG was involved in the conception and design of the research idea, coordination and acquisition of the data, analyses and interpretation of the data, and in drafting, reviewing and revising the manuscript. MD was involved with the conception and design of the research idea, coordination and acquisition of the data, interpretation of the data, and in reviewing the final manuscript. AO was involved with the conception and design of the research idea, coordination and the acquisition of the data, interpretation of the data, and in reviewing the final manuscript. DES was involved in the conception and design of the research idea, interpretation of the data and reviewing the final manuscript. All authors read and approved the final manuscript.

## Pre-publication history

The pre-publication history for this paper can be accessed here:


